# Working Memory States Modulate Arithmetic Estimation Strategy Processing: Evidence from Visual and Articulatory Interference

**DOI:** 10.3390/bs16091588

**Published:** 2026-09-07

**Authors:** Mengmeng Li, Yun Pan, Di Zhang, Shisan Qi

**Affiliations:** 1 School of Psychology, Guizhou Normal University, Guiyang 550025, China; mml890321@outlook.com (M.L.); panyun129@163.com (Y.P.); zdavatar@gznu.edu.cn (D.Z.); 2 School of Teacher Education, Zunyi Normal University, Zunyi 563006, China; 3 Guizhou Education University, Guiyang 550018, China; 4 School of Psychology, Inner Mongolia Normal University, Hohhot 010022, China

**Keywords:** working memory states, arithmetic estimation strategy processing, online state, offline state, serial probe task, estimation strategy difficulty sequence, visual interference, articulatory interference

## Abstract

Arithmetic estimation requires maintaining numerical information while applying rule-based procedures for approximate calculation, yet it remains unclear whether such estimation strategy processing depends on the working memory state in which task-relevant information is maintained. This study examined whether operationally defined online and offline working memory states modulate arithmetic estimation strategy processing, and whether such modulation varies with estimation strategy difficulty sequence and interference condition. Through three behavioral experiments, participants completed a serial probe task requiring estimation strategy consistency judgments for rounding down, rounding up, and mixed rounding strategies. Experiment 1 manipulated presentation duration and revealed distinct temporal profiles: online state performance was highest at the shortest duration and declined thereafter, whereas offline state performance improved and then stabilized. Experiment 2 showed that visual interference did not produce a uniform impairment, but selectively modulated online state estimation strategy judgments in a difficulty-sequence-dependent manner. Experiment 3 showed that articulatory interference produced broader disruption, impairing offline state performance across sequence conditions and reducing online state performance in the difficult–simple sequence. These findings are consistent with the view that arithmetic estimation strategy performance varies with operationally defined working memory representational state, difficulty sequence context, and the task demands imposed by different interference conditions.

## 1. Introduction

Arithmetic strategies refer broadly to the procedures individuals use to solve numerical problems, including retrieval, decomposition, counting, algorithmic calculation, and estimation. Within this broad domain, arithmetic estimation strategies are a specific class of procedures used to generate or evaluate approximate rather than exact outcomes. The present study focuses specifically on arithmetic estimation strategy processing.

Arithmetic estimation is an important component of numerical cognition, particularly when exact calculation is unnecessary or cognitively costly. Unlike exact calculation, estimation relies on simplified numerical transformations and strategy-based processing (Ashcraft, 1992; Reys, 1984; Sekeris et al., 2021). Estimation performance therefore depends on the use of strategies suited to problem characteristics and task demands (Lemaire & Siegler, 1995; Siegler & Lemaire, 1997; Lemaire et al., 2000, 2004; Lemaire & Lecacheur, 2002).

The present study examines estimation strategy information using rounding strategies, which provide clear operational distinctions in processing demands. Rounding down involves rounding both operands down to the nearest decade, rounding up involves rounding both operands up, and mixed rounding requires rounding one operand up and the other down (Imbo et al., 2007; Lemaire et al., 2004; Uittenhove & Lemaire, 2012). Rounding down is generally less demanding, whereas rounding up and mixed rounding require greater maintenance and control of estimation rules (Imbo et al., 2007; Poloczek et al., 2022). These distinctions provide the basis for examining arithmetic estimation strategy processing in the present study.

Strategy sequential difficulty (SSD) effects provide a useful framework for examining how the difficulty of one arithmetic strategy episode relates to subsequent processing. Previous studies have shown that performing a difficult strategy can impair performance on the following strategy, possibly reflecting lingering demands on retrieval, monitoring, or executive control (Uittenhove & Lemaire, 2012, 2013; Hinault et al., 2014, 2017). In the present study, this logic was extended to arithmetic estimation by comparing difficult–simple and simple–difficult problem sequences. Because these cross-difficulty sequences vary in both preceding and current problem difficulty, sequence effects are treated as contextual differences in processing demands rather than as a pure measure of residual load.

Working memory temporarily maintains and manipulates information required for ongoing cognition and is closely involved in arithmetic processing (A. D. Baddeley & Hitch, 1974; Seitz & Schumann-Hengsteler, 2000; A. Baddeley, 2000, 2012). In arithmetic estimation, it supports the maintenance of operands, estimation rules, and intermediate information while controlled transformations are performed (Logie et al., 1994). Meta-analytic and empirical evidence consistently link working memory to mathematical performance, particularly when controlled strategy execution rather than direct fact retrieval is required (Friso van den Bos et al., 2013; Hubber et al., 2014; Peng et al., 2016; Raghubar et al., 2010; Chen & Bailey, 2021;Cragg et al., 2017; Hecht, 2002; Imbo & Vandierendonck, 2007). Working memory updating has also been associated with computational estimation performance and strategy use (Hammerstein et al., 2019, 2021).

However, previous arithmetic and estimation research has largely examined working memory in terms of capacity or component resources. Less attention has been given to whether estimation strategy information is processed differently depending on its current priority and accessibility within working memory. This distinction is important because information required for an immediate judgment may not be represented in the same functional state as information that must be retained for later use.

State-based accounts propose that maintained information can occupy functionally distinct representational states (Nee & Jonides, 2013). Information in an online state or active state is prioritized for current use, directly accessible, and closely coupled with the focus of attention (Gazzaley & Nobre, 2012; Kreither et al., 2022). Information in an offline or latent state is no longer prioritized for immediate action but remains available for later retrieval or reactivation (Cowan, 1999, 2005; LaRocque et al., 2014; Rose et al., 2016; Lewis-Peacock et al., 2012; Trübutschek et al., 2017; Barbosa et al., 2020; de Vries et al., 2020; Oberauer, 2002). The terms “online” and “offline” states should therefore be interpreted functionally rather than as direct evidence for distinct neural storage mechanisms (Postle, 2006; D’Esposito & Postle, 2015). In the present study, they refer to differences in attentional priority and immediate accessibility. This functional distinction provides a framework for examining how estimation strategy information is maintained and accessed during ongoing processing without assuming a specific underlying neural mechanism. (Beukers et al., 2021; Oberauer & Awh, 2022)

Linking arithmetic estimation strategy processing to working memory state raises a more specific question than whether estimation simply depends on working memory capacity. Estimation requires numerical and strategy information to remain available while nonautomatic transformations and comparisons are performed, and working memory updating has been associated with computational estimation strategy processing (Hammerstein et al., 2019, 2021). In a sequential estimation task, currently relevant information may be prioritized for immediate processing, whereas earlier information must remain accessible for subsequent judgments. Thus, estimation strategy processing may vary with the current priority of information in working memory and with the difficulty sequence in which that information is processed.

Interference provides a further approach to examining how working memory accessibility relates to arithmetic estimation strategy processing. Previous research has shown that concurrent processing demands can influence the maintenance of information in working memory by consuming limited attentional and domain-specific resources (Barrouillet et al., 2007; Camos et al., 2009; Wei & Zhang, 2025). In particular, verbal information maintenance may depend on articulatory rehearsal and attentional refreshing, and additional speech production demands can interfere with these maintenance processes (Salamé & Baddeley, 1982; A. Baddeley, 2000; Camos et al., 2009). Moreover, arithmetic operations differ in their reliance on representational codes. According to the triple code framework, multiplication fact retrieval relies relatively strongly on verbal representations, whereas other operations may rely more on magnitude-based processing (Dehaene, 1992; Dehaene & Cohen, 1995; Dehaene et al., 1999, 2003). Consistent with this view, experimental studies have shown that multiplication fact retrieval is particularly associated with phonological processing and can be affected by concurrent articulation (Lee & Kang, 2002; Moeller et al., 2011).

Because arithmetic estimation involves visual symbolic information as well as numerical and verbal processing, task-irrelevant arithmetic input may influence strategy information differently depending on the cognitive demands it imposes. In the present study, visual arithmetic interference was introduced by presenting an irrelevant multiplication distractor, whereas articulatory interference was implemented by requiring participants to read aloud the visually presented distractor. The latter manipulation was designed to introduce additional articulatory processing demands rather than to represent a pure phonological interference condition. Comparing these two forms of interference allowed us to examine whether estimation strategy processing across working memory conditions is differentially modulated by distinct types of concurrent processing demands.

The present study examined whether arithmetic estimation strategy processing varies with the functional priority of information in working memory, and whether these patterns are further influenced by difficulty sequence and interference. Previous research has distinguished prioritized information within the focus of attention from information maintained at a lower priority but remaining accessible for subsequent use (Oberauer, 2002; Olivers et al., 2011; LaRocque et al., 2014; Myers et al., 2017). Building on this framework, we used a serial probe estimation strategy consistency task involving rounding down, rounding up, and mixed rounding strategies. The second presented problem, which was probed first, was operationalized as the online state, whereas the first presented problem, which was retained during subsequent processing and probed later, was operationalized as the offline state. This serial probe manipulation provides a functional operationalization of relative priority and accessibility, although representational state is not isolated from the accompanying differences in serial position and probe order. Across three experiments, we examined how performance in these operationalized conditions varied with presentation duration, difficulty sequence, and interference. Experiment 1 examined whether online and offline states showed different performance patterns across presentation durations. Experiment 2 introduced visually presented arithmetic distractors to examine the effects of visual interference across working memory states and difficulty sequences. Experiment 3 additionally required participants to read the arithmetic distractor aloud, allowing us to examine the effects of additional articulatory demands. Together, the experiments tested whether arithmetic estimation strategy processing varies with working memory priority and with the demands imposed by difficulty sequence and interference.

## 2. Experiment 1

### 2.1. Experiment 1 Overview and Hypotheses

Experiment 1 examined whether arithmetic estimation strategy performance differed between items operationalized as online and offline states under different presentation durations. A serial probe paradigm was used in which two multiplication problems were presented sequentially within each trial. The second presented problem (M2), which was probed first, was operationalized as the online state, whereas the first presented problem (M1), which had to be retained while M2 was processed and was probed later, was operationalized as the offline state.

Presentation duration was manipulated at three levels: 1000 ms, 1500 ms, and 2000 ms. We predicted that performance for the online and offline states would vary as a function of presentation duration. Specifically, if additional processing time differentially influenced the accessibility of the two items, the magnitude of the online and offline state performance difference should change across presentation durations. This design therefore allowed us to examine whether the two operationalized working memory states showed different performance patterns as the time available for processing increased.

### 2.2. Method

#### 2.2.1. Participants

An a priori power analysis was conducted before data collection using G*Power 3.1.9.7 to determine the required sample size for Experiment 1. The analysis used the F-test family with a repeated-measures ANOVA (within-factors), specifying an effect size of f = 0.25, α = 0.05, statistical power (1 − β) = 0.90, a correlation of 0.50 among repeated measures, and a nonsphericity correction of ε = 1.00. The analysis indicated a minimum required sample size of 36 participants. Because the experimental materials and estimation strategy judgments imposed relatively high task demands upon some participants, we recruited more participants than this minimum requirement in advance to ensure that a sufficient number of valid datasets would remain after application of the predefined exclusion criteria and any unexpected data loss.

A total of 96 university students were recruited for Experiment 1. All participants were aged 18 years or older, right-handed, and reported normal or corrected-to-normal vision, with no amblyopia or color blindness. The exclusion criteria were specified a priori, before data analysis. Because both the estimation strategy judgment and equation judgment tasks required binary same/different responses, participants were retained only when their accuracy was at least 0.50; this criterion was also applied separately to online and offline state responses to ensure that performance in neither condition was at or below chance level. Thirty participants were excluded according to this performance criterion. An additional 13 participants were excluded because they completed an incorrect version of the experimental program. These two exclusion categories did not overlap. The final sample therefore consisted of 53 participants (14 males and 39 females), exceeding the minimum sample size indicated by the a priori power analysis.

All participants provided written informed consent before the experiment and were informed of the experimental procedure, potential risks, task requirements, and their right to withdraw at any time. The study was approved by the university ethics committee.

#### 2.2.2. Materials

The stimuli consisted of two-digit multiplication problems. The materials were constructed according to the following constraints: (a) no operand contained repeated digits (e.g., 44); (b) no operand ended in 0 or 5 (e.g., 20 or 25); (c) reversed operand pairs were not used across problems (e.g., 43 × 82 and 82 × 43); (d) in half of the problems, the first operand was larger than the second operand, whereas the reverse was true in the other half; (e) no operand could be rounded to 0, 10, or 100, which excluded operands with tens digits of 1 or 9; and (f) within the same equation, the two operands did not share the same tens or units digit (e.g., 43 × 41 or 32 × 82). These constraints were used to reduce superficial digit matching strategies and to encourage estimation strategy level processing.

The experiment focused on three commonly studied arithmetic estimation strategies: rounding down, rounding up, and mixed rounding. Rounding down refers to rounding both operands down to the nearest decade; rounding up refers to rounding both operands up; and mixed rounding refers to rounding one operand up and the other down (Lemaire et al., 2004; Imbo et al., 2007; Uittenhove & Lemaire, 2012). These three strategies served as the core categories for the estimation strategy consistency judgment task. For each equation, the optimal estimation strategy was defined as the strategy whose estimated result produced the smallest deviation from the exact result. Thus, the task indexed the processing of estimation strategy information rather than the retrieval of exact products.

#### 2.2.3. Apparatus and Software

The experiment was programmed and presented using PsychoPy 2024.1.0. Stimuli were displayed on a 15.6-inch monitor with a resolution of 1920 × 1080 pixels. Responses were collected using a standard keyboard.

#### 2.2.4. Design

Experiment 1 employed a 2 × 3 within-subjects design, with working memory state (online state vs. offline state) and presentation duration (1000 ms, 1500 ms, and 2000 ms) as within-subject factors. The second presented problem (M2), which was probed first, was operationalized as the online state, whereas the first presented problem (M1), which was probed after M2, was operationalized as the offline state. The dependent variable was accuracy in the estimation strategy consistency judgment task.

The formal experiment consisted of 90 trial sets, divided into three presentation duration blocks of 30 trial sets each. Within a given block, both M1 and M2 were presented for the same duration (1000 ms, 1500 ms, or 2000 ms). Each trial set contained two memory problems (M1 and M2), followed by two corresponding probes (T2 and T1), yielding two probe-level responses per trial set and 180 probe-level responses in total. The order of the three duration blocks was randomized independently for each participant. At the start of the experiment, PsychoPy generated a random sequence of the three block identifiers, which determined the order in which the short, medium, and long duration blocks were presented.

Estimation strategy judgment trials and equation judgment trials each accounted for 50% of the trials and were randomly interleaved within each block. Within both judgment types, same and different responses were equally represented. In estimation strategy judgment trials, a same probe corresponded to the same rounding strategy category as the memorized equation. For a different probe, one of the other two strategy categories was selected at random. Thus, for example, when the memorized equation was associated with a rounding down strategy, a different probe was associated with either a rounding up or mixed rounding strategy. Transitions among the three strategy categories were randomized, and rounding down, rounding up, and mixed rounding trials were approximately balanced within each presentation duration block.

In equation judgment trials, a same probe was identical to the corresponding memorized equation, whereas a different probe was created by altering one or both operands at random while retaining the stimulus construction constraints described above. Equation judgment trials were randomly interleaved with estimation strategy judgment trials to discourage participants from selectively retaining only the unit digit information sufficient for identifying the rounding strategy. Because participants could not predict which type of judgment would be required, the equation judgment trials encouraged them to maintain more complete equation-specific information rather than relying solely on strategy-relevant digit features.

Before the formal experiment, participants completed 36 practice trials with feedback and were required to achieve at least 80% accuracy to ensure that they understood the task and response requirements.

#### 2.2.5. Procedure

Participants first provided written informed consent and received detailed task instructions. Before the formal experiment, they completed a strategy training phase in which the three estimation strategies (rounding down, rounding up, and mixed rounding) were explained with examples. Participants were instructed to determine the optimal strategy for each multiplication problem according to the strategy that produced the smallest deviation between the estimated and exact results. They then completed 36 practice trials. No trial-by-trial feedback was provided during practice; after completing the practice trials, participants were informed whether they had reached the required accuracy criterion of 80%. Participants who did not reach this criterion repeated the practice phase before proceeding to the formal experiment.

Each formal trial began with a fixation cross presented at the center of the screen for 500 ms. The first two-digit multiplication problem (M1) was then presented for 1000 ms, 1500 ms, and 2000 ms, depending on the presentation duration block, followed by a 500 ms black and white mosaic mask. The second two-digit multiplication problem (M2) was presented for the same duration and was followed by an identical 500 ms mosaic mask. The masks were used to reduce visual persistence of the preceding multiplication problems. Two probes were then presented sequentially in the fixed order T2 followed by T1: T2 corresponded to M2, whereas T1 corresponded to M1. Thus, M2/T2 constituted the item operationalized as the online state, and M1/T1 constituted the item operationalized as the offline state.

Two types of probe judgment were randomly interleaved. In estimation strategy judgment trials, participants determined the optimal rounding strategy for the memorized multiplication problem and judged whether the probe equation required the same optimal strategy. Thus, for T2, participants judged whether the optimal estimation strategy for T2 was the same as that for M2; for T1, they judged whether the optimal strategy for T1 was the same as that for M1. Importantly, participants were not asked to report which of the three strategies they had identified; instead, they made a binary same or different judgment regarding strategy consistency. In equation judgment trials, participants judged whether T2 was identical to M2 and whether T1 was identical to M1, again using a binary same or different response.

Each probe remained on the screen until a response was made or until the 5000 ms response deadline was reached. A response made before the deadline immediately initiated the next probe; thus, T1 followed T2 without an additional mask, fixation, or interstimulus interval. If no response was made within 5000 ms, the trial advanced automatically and the missing response was coded as incorrect. Response key mapping was counterbalanced across participants: for half of the participants, the F key indicated same and the J key indicated different, whereas the mapping was reversed for the remaining participants.

#### 2.2.6. Data Analysis

All analyses were conducted in Python 3.9.13 using pandas 1.4.4, NumPy 1.21.5, SciPy 1.9.1, statsmodels 0.13.2, and patsy 0.5.2. Trial-level accuracy in the estimation strategy judgment task was analyzed using a population-averaged generalized estimating equation (GEE) with a binomial distribution and logit link. Working memory state (online state vs. offline state), presentation duration (1000 ms, 1500 ms, and 2000 ms), and their interaction were included as fixed effects, with the participant treated as the clustering variable and an exchangeable working correlation structure used to account for repeated observations.

Omnibus effects were evaluated using Type III Wald χ^2^ tests. Significant interactions were followed by pairwise contrasts, which were expressed as odds ratios (ORs) with 95% confidence intervals. Holm correction was applied to post hoc comparisons. Equation judgment accuracy was summarized descriptively and was not included in the primary inferential model.

### 2.3. Results

All 53 participants contributed 90 formal trial sets, yielding 4770 estimation strategy judgments and 4770 equation judgments. For the estimation strategy judgment task, model-estimated accuracy in the online state was 0.879, 95% CI [0.845, 0.907], at 1000 ms; 0.808, 95% CI [0.764, 0.844], at 1500 ms; and 0.769, 95% CI [0.733, 0.801], at 2000 ms. Corresponding estimates in the offline state were 0.756, 95% CI [0.726, 0.784]; 0.806, 95% CI [0.767, 0.840]; and 0.784, 95% CI [0.748, 0.815], respectively.

Type III Wald tests revealed a significant marginal effect of working memory state, Wald χ^2^(1) = 6.42, *p* = 0.011, marginal OR = 1.29, 95% CI [1.06, 1.58], and a significant effect of presentation duration, Wald χ^2^(2) = 16.86, *p* < 0.001. More importantly, the working memory state × presentation duration interaction was significant, Wald χ^2^(2) = 26.64, *p* < 0.001, indicating that the online and offline state accuracy difference varied across presentation durations.

Follow-up contrasts showed that online state accuracy was higher than offline state accuracy at 1000 ms, OR = 2.35, 95% CI [1.73, 3.19], Holm-adjusted *p* < 0.001. The two conditions did not differ at 1500 ms (OR = 1.01, 95% CI [0.72, 1.40], adjusted *p* = 0.964), or at 2000 ms (OR = 0.92, 95% CI [0.73, 1.15], adjusted *p* = 0.920).

**Figure 1:**
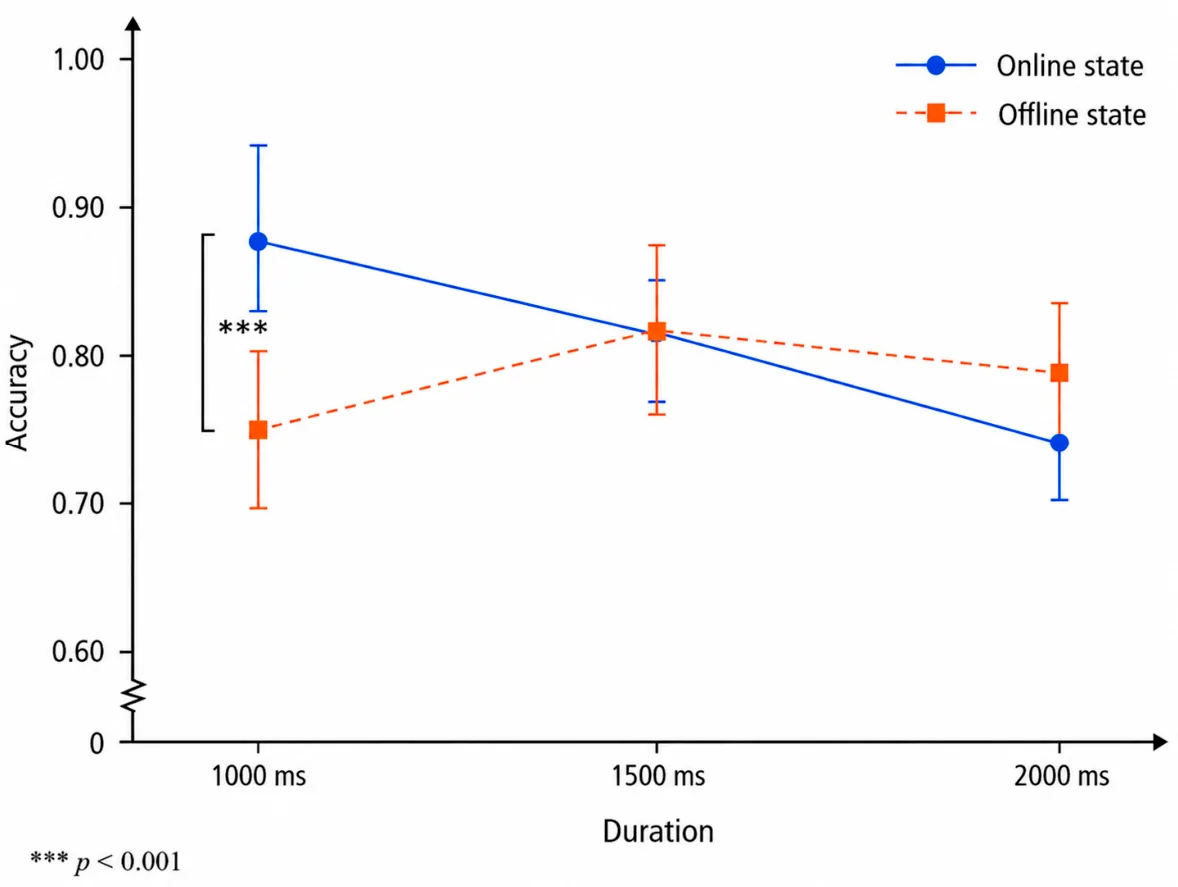
Estimation strategy judgment accuracy across operationalized online and offline working memory states under different presentation durations.

Presentation duration significantly affected accuracy within the online state, Wald χ^2^(2) = 29.47, *p* < 0.001. Relative to 1000 ms, the odds of a correct online state response were lower at 1500 ms (OR = 0.58, 95% CI [0.41, 0.81], adjusted *p* = 0.003) and at 2000 ms (OR = 0.46, 95% CI [0.33, 0.62], adjusted *p* < 0.001). Online state accuracy was also lower at 2000 ms than at 1500 ms, OR = 0.79, 95% CI [0.66, 0.94], adjusted *p* = 0.009. Presentation duration also affected accuracy within the offline state, Wald χ^2^(2) = 6.71, *p* = 0.035. Offline state accuracy was higher at 1500 ms than at 1000 ms, OR = 1.34, 95% CI [1.07, 1.69], adjusted *p* = 0.033. The difference between 2000 ms and 1000 ms was not significant (OR = 1.17, 95% CI [0.98, 1.40], adjusted *p* = 0.169), nor was the difference between 2000 ms and 1500 ms, OR = 0.87, 95% CI [0.71, 1.07], adjusted *p* = 0.183. Online and offline state contrasts across presentation durations are shown in Figure 1.

Equation judgment accuracy was also examined descriptively as a validation measure. Collapsed across presentation durations, participant-level mean accuracy was 0.855 (SD = 0.096) for online state probes and 0.753 (SD = 0.104) for offline state probes. Across both judgment types, all retained participants exceeded 0.50 accuracy overall and separately for online and offline state probes. These descriptive results indicate that participants retained equation-specific information at above chance levels in both operationalized conditions, although they do not establish that participants relied exclusively on complete equation representations rather than partial digit information.

### 2.4. Discussion

Experiment 1 examined how arithmetic estimation strategy performance varied across presentation durations for items operationalized as online and offline working memory states in the serial probe task. The significant interaction between working memory state and presentation duration revealed different temporal performance patterns across the two conditions. At 1000 ms, accuracy was significantly higher for the online than for the offline state, whereas the two states, as operationalized in the present task, no longer differed at 1500 ms or 2000 ms. Within the online state, accuracy decreased as presentation duration increased. In contrast, offline state accuracy improved from 1000 ms to 1500 ms and did not change significantly thereafter. These findings suggest that arithmetic estimation strategy information associated with the operationalized online and offline states showed different patterns of accessibility as a function of processing time.

The online state pattern is broadly consistent with working memory state accounts emphasizing the privileged accessibility of information currently prioritized for use (Cowan, 2005; Oberauer, 2002; Myers et al., 2017). Functionally, the online state refers to information that is currently prioritized and readily accessible for ongoing processing. In the present task, M2 was the most recently presented item and was probed first, thereby serving as the operationalized online state. Its advantage at the shortest presentation duration is therefore compatible with the high accessibility generally associated with prioritized working memory state. The subsequent decline in online state performance suggests, however, that this initial advantage was time-sensitive rather than sustained across longer processing intervals. Previous work has similarly emphasized that priority in working memory is dynamic rather than fixed, and that the accessibility of currently relevant information can vary as task demands evolve (Olivers et al., 2011; Myers et al., 2017). One possible interpretation is that the online state advantage primarily reflects a transient prioritization benefit rather than a stable superiority of the representation itself. As processing time increases, attention may become less exclusively concentrated on the most recently presented item and more broadly distributed across the information that must be retained for subsequent judgments. From this perspective, a longer processing time could reduce the relative advantage associated with immediate priority, even though the information itself remains available. This interpretation is compatible with contemporary views that working memory state priority can be dynamically reconfigured according to changing task demands rather than remaining fixed throughout a trial (Olivers et al., 2011; Myers et al., 2017).

A second, complementary possibility is that prolonged presentation permits more extensive evaluation of numerical and strategy-related information. Arithmetic estimation requires not only perceptual encoding of the operands but also identification and maintenance of the relevant transformation rule. Additional processing time may therefore increase the amount of strategy-related information being evaluated and maintained, potentially increasing competition among task-relevant representations rather than simply strengthening the currently prioritized item. The present experiment was not designed to distinguish attentional redistribution from changes in encoding or strategy evaluation, but the decline in online state performance suggests that greater processing time does not necessarily produce a monotonic benefit for information that is already prioritized. More broadly, this pattern indicates that the behavioral advantage associated with online state accessibility may depend on when the information is probed and the processing demands imposed before retrieval.

The offline state pattern differed from that observed for the online state. Functionally, the offline state refers to information that is no longer prioritized for immediate processing but remains available for subsequent use. In the present task, M1 had to be retained while M2 was processed and was subsequently probed as T1, thereby serving as the operationalized offline item. Offline state accuracy was lower at 1000 ms but increased at 1500 ms, with no further significant change at 2000 ms. This pattern suggests that later access to the first presented problem benefited from additional processing time. Computational estimation requires numerical and strategy information to be maintained while an appropriate estimation strategy is identified, and previous studies have shown that working memory state updating and presentation duration can influence estimation strategy processing (Hammerstein et al., 2019, 2021, 2022). From a state-based perspective, the present pattern is compatible with the proposal that information outside the current focus of attention can remain available for subsequent use (Oberauer, 2002; Myers et al., 2017). At the same time, additional processing time may also have strengthened initial encoding or facilitated identification of the relevant estimation strategy. More specifically, the improvement from 1000 ms to 1500 ms may reflect stronger initial encoding, more complete identification of the relevant estimation strategy, or greater readiness of the retained information for later retrieval.

The absence of a further significant improvement between 1500 ms and 2000 ms additionally suggests that the benefit associated with increased processing time may reach a functional limit. Once sufficient numerical and strategy-related information has been encoded to support the subsequent judgment, further exposure may provide relatively little additional benefit. From this perspective, the convergence of online and offline performance at 1500 ms may reflect two complementary temporal changes: the initial accessibility advantage of the online state decreased, whereas the later accessibility of the offline state improved. Such convergence is therefore compatible with a redistribution in the relative accessibility of the two task-defined representations rather than a discrete transition from one storage state to another. The improvement in offline state performance should therefore not be taken as direct evidence of a discrete transition into a distinct offline state.

This temporal pattern also has broader implications for understanding the contribution of working memory to arithmetic strategy processing. Previous arithmetic research has primarily emphasized the amount of working memory state capacity available or the particular working memory state components involved in strategy execution. The present findings suggest that when information is accessed and how it is prioritized within an ongoing processing sequence may also be important. In other words, arithmetic strategy information may support different levels of performance depending on its current functional accessibility within the task. This extends working memory state approaches to computational estimation by suggesting that strategy processing is sensitive not only to overall resource availability but also to the temporal organization and accessibility of maintained information.

The online and offline states in the present study should therefore be understood as functionally operationalized working memory states rather than as directly measured storage states. Because M1 and M2 also differed in serial position and probe order, recency, retention interval, and the intervening processing of M2 may have contributed to the observed differences. These factors do not preclude a state-based interpretation, but they limit the extent to which the behavioral effects can be attributed exclusively to representational working memory state. Accordingly, the present findings are best interpreted as showing that task-defined differences in priority and accessibility interact with processing time during arithmetic estimation strategy judgments, rather than as demonstrating distinct underlying storage mechanisms.

Importantly, online and offline state performance converged at 1500 ms, providing a useful baseline for the subsequent experiments. At this presentation duration, accuracy was closely matched across the two operationalized working memory states, reducing the likelihood that subsequent differences would simply reflect an initial performance imbalance. Therefore, Experiment 2 fixed the presentation duration at 1500 ms and examined whether visually presented arithmetic interference and difficulty sequence further modulated estimation strategy processing in the online and offline states.

## 3. Experiment 2

### 3.1. Experiment 2 Overview and Hypotheses

Experiment 2 examined whether visually presented arithmetic distractors modulated arithmetic estimation strategy processing under different working memory states. In Experiment 1, online and offline working memory states showed distinct temporal profiles, and their performance converged when the presentation duration was 1500 ms. Therefore, in Experiment 2, the presentation duration of both arithmetic problems was fixed at 1500 ms, and a visual interference manipulation was introduced before the probe phase.

State-based accounts of working memory suggest that online and offline state representations may differ in their susceptibility to external distraction. Online state representations are highly accessible and prioritized for current processing, but this accessibility may also make them more exposed to task-irrelevant perceptual input (LaRocque et al., 2014; Zokaei et al., 2014). By contrast, offline state or activity-silent representations are assumed to be maintained outside the current focus of attention and may therefore be relatively protected from immediate perceptual interference (Mongillo et al., 2008; Stokes, 2015; Wolff et al., 2017; J. Zhang et al., 2022).

The present experiment introduced visually presented multiplication distractors to test whether external visual symbolic information would disrupt estimation strategy consistency judgments. Because arithmetic estimation strategy processing involves not only the perceptual encoding of equations, but also numerical comparison, estimation rule maintenance, and goal-directed control, visual interference was not expected to produce a necessarily uniform disruptive effect. Instead, we examined whether its influence would depend on the working memory state of the probed item and the difficulty sequence of the two estimation problems.

### 3.2. Method

#### 3.2.1. Participants

An a priori power analysis was conducted before data collection using G*Power 3.1.9.7 to determine the required sample size for Experiment 2. The analysis used the F-test family with a repeated-measures ANOVA (within-factors) as the design-level framework for the 2 × 2 × 2 within-subject design. The parameters were an effect size of f = 0.25, α = 0.05, statistical power (1 − β) = 0.90, a correlation of 0.50 among repeated measures, and a nonsphericity correction of ε = 1.00. The analysis indicated a minimum required sample size of 20 participants. Because the experimental task was relatively demanding and some data loss was anticipated after application of the predefined performance criteria, we recruited more participants than this minimum requirement to ensure an adequate final sample.

A total of 47 university students were recruited for Experiment 2. All participants were aged 18 years or older, right-handed, reported normal or corrected-to-normal vision, and had no amblyopia or color blindness. None had participated in Experiment 1. The exclusion criteria were specified a priori, before data analysis, and were identical to those used in Experiment 1. Because both the estimation strategy judgment and equation judgment tasks required binary responses, participants were retained only when accuracy was at least 0.50 overall and separately for the online and offline states responses. A total of 13 participants did not meet these predefined performance criteria and were excluded. No participants were excluded for other reasons. The final sample consisted of 34 participants (7 males and 27 females), exceeding the minimum sample size indicated by the a priori power analysis. The G*Power analysis was based on a repeated-measures ANOVA framework and therefore served as a design-level estimate of the required sample size. It did not directly model the trial-level binomial GEE used for the primary analyses or specifically estimate statistical power for the three-way interaction. The power analysis should therefore be interpreted as an a priori design-level sample-size reference rather than a direct power analysis of the final GEE model.

All participants provided written informed consent before the experiment and were informed of the experimental procedure, potential risks, task requirements, and their right to withdraw at any time. The study was approved by the university ethics committee.

#### 3.2.2. Materials

The stimuli were two-digit multiplication problems constructed according to the same general constraints as in Experiment 1. Problem difficulty was recomputed from the unit digits of the two operands. A problem was classified as simple when the unit digits of both operands were smaller than 5. A problem was classified as difficult when both unit digits were larger than 5, or when one unit digit was larger than 5 and the other was smaller than 5. Based on the difficulty of M1 and M2, two cross-difficulty sequence types were defined: difficult–simple and simple–difficult. In the present context, “difficult” and “simple” refer to experimentally defined estimation processing difficulty, not to participants’ spontaneous strategy choices.

#### 3.2.3. Apparatus and Software

The experiment was programmed and presented using PsychoPy 2024.1.0. The apparatus and software were identical to those used in Experiment 1.

#### 3.2.4. Design

Experiment 2 employed a 2 × 2 × 2 within-subjects design, with working memory state (online state vs. offline state), difficulty sequence (difficult–simple vs. simple–difficult), and visual interference (interference vs. no interference) as within-subject factors. The second presented problem (M2), which was probed first, was operationalized as the online state, whereas the first presented problem (M1), which was probed after M2, was operationalized as the offline state. The dependent variable was accuracy in the estimation strategy consistency judgment task.

The experiment consisted of two blocks, with 60 trial sets in each block, resulting in 120 trial sets per participant. Each trial set followed the sequence M1 → M2 → T2 → T1 and generated two probe-level responses, yielding 240 probe-level responses per participant. Both M1 and M2 were presented for 1500 ms, as determined by the results of Experiment 1.

Difficulty sequence was defined according to the difficulty of the two estimation problems: difficult–simple sequences consisted of a difficult M1 followed by a simple M2, whereas simple–difficult sequences consisted of a simple M1 followed by a difficult M2. Visual interference and no-interference trials were equally represented and randomly intermixed across participants. The three factors were fully randomized at the trial level.

The two judgment types were randomly intermixed within each block, with estimation strategy judgment trials accounting for 70% of trials and equation judgment trials accounting for 30% of trials. The construction of same/different trials and the generation of estimation strategy and equation judgments followed the procedures described in Experiment 1. Briefly, same and different responses were equally represented, and different strategy trials were generated by randomly selecting one of the alternative rounding strategies. Equation judgment trials were constructed by presenting either identical equations or equations with randomly altered operands while maintaining the stimulus constraints described previously. The three rounding strategies were approximately balanced across trials. The visual distractors followed the same stimulus construction criteria as the multiplication problems used in Experiment 1.

#### 3.2.5. Procedure

The practice phase was identical to that in Experiment 1.

Each formal trial began with a fixation cross presented at the center of the screen for 500 ms. The first multiplication problem (M1) was then presented for 1500 ms, followed by a 500 ms black and white mosaic mask. The second multiplication problem (M2) was subsequently presented for 1500 ms and followed by another identical 500 ms mask. The two problems were therefore encoded under the same presentation duration as determined by Experiment 1.

After the second mask, the trial diverged according to the visual interference condition. In the visual interference condition, an irrelevant two-digit multiplication problem was presented at the center of the screen for 1500 ms. Participants were instructed to simply view the distractor and were not required to make any response. The distractor equations followed the same stimulus construction criteria as the experimental multiplication problems and were randomly generated while avoiding identity with M1, M2, T1, or T2 within the same trial. In the no-interference condition, a blank screen was presented for 1500 ms instead, thereby equating the duration between the second mask and the probe phase across conditions.

Participants then completed two sequential probe judgments in the fixed order T2 followed by T1. T2 corresponded to M2 and was used to assess the item operationalized as the online state, whereas T1 corresponded to M1 and was used to assess the item operationalized as the offline state. The probe procedure and response requirements were identical to those in Experiment 1. Briefly, in estimation strategy judgment trials, participants judged whether the optimal estimation strategy of the probe equation was consistent with that of the corresponding memorized equation. In equation judgment trials, participants judged whether the probe equation was identical to the corresponding memorized equation. Responses were made using a binary same/different judgment.

Probe equations remained on the screen until a response was made or until the 5000 ms response deadline was reached. Responses exceeding this deadline were coded as incorrect. Participants completed two blocks of trials, with the order of trials randomized within blocks. Difficulty sequence (difficult–simple vs. simple–difficult) and visual interference condition (interference vs. no interference) were randomly intermixed across participants.

#### 3.2.6. Data Analysis

All analyses were conducted in Python 3.9.13 using pandas 1.4.4, NumPy 1.21.5, SciPy 1.9.1, statsmodels 0.13.2, and patsy 0.5.2. Trial-level accuracy in the estimation strategy judgment task was analyzed using a population-averaged generalized estimating equation (GEE) with a binomial distribution and logit link. Working memory state (online state vs. offline state), difficulty sequence (simple–difficult vs. difficult–simple), visual interference (interference vs. no interference), and their interactions were included as fixed effects, with the participant treated as the clustering variable and an exchangeable working correlation structure used to account for repeated observations.

Omnibus effects were evaluated using Type III Wald χ^2^ tests. Significant interactions were followed by pairwise contrasts, which were expressed as odds ratios (ORs) with 95% confidence intervals. Holm correction was applied to post hoc comparisons. Equation judgment accuracy was summarized descriptively and was not included in the primary inferential model.

### 3.3. Results

A total of 34 participants were included in the final analysis after applying the accuracy screening criterion. Model-estimated accuracy was calculated as a function of working memory state, difficulty sequence, and visual interference.

For the online state, accuracy in the difficult–simple sequence was 0.805 (95% CI [0.757, 0.846]) without visual interference and 0.735 (95% CI [0.654, 0.803]) with visual interference. In the simple–difficult sequence, online state accuracy was 0.618 (95% CI [0.552, 0.679]) without interference and increased to 0.776 (95% CI [0.716, 0.827]) with interference. For the offline state, accuracy remained relatively stable across interference conditions. Specifically, in the difficult–simple sequence, offline state accuracy was 0.739 (95% CI [0.647, 0.814]) without interference and 0.735 (95% CI [0.673, 0.789]) with interference. In the simple–difficult sequence, offline state accuracy was 0.725 (95% CI [0.652, 0.789]) without interference and 0.731 (95% CI [0.664, 0.789]) with interference.

The Type III Wald tests showed no significant main effect of working memory state, Wald χ^2^(1) = 0.11, *p* = 0.736, a significant main effect of difficulty sequence, Wald χ^2^(1) = 4.19, *p* = 0.041, and no significant main effect of visual interference, Wald χ^2^(1) = 1.29, *p* = 0.257. The working memory state × difficulty sequence interaction was not significant, Wald χ^2^(1) = 3.24, *p* = 0.072, nor was the working memory state × visual interference interaction, Wald χ^2^(1) = 0.86, *p* = 0.355. However, the difficulty sequence × visual interference interaction was significant, Wald χ^2^(1) = 8.27, *p* = 0.004. Importantly, these effects were qualified by a significant three-way interaction among working memory state, difficulty sequence, and visual interference, Wald χ^2^(1) = 9.11, *p* = 0.003.

**Figure 2:**
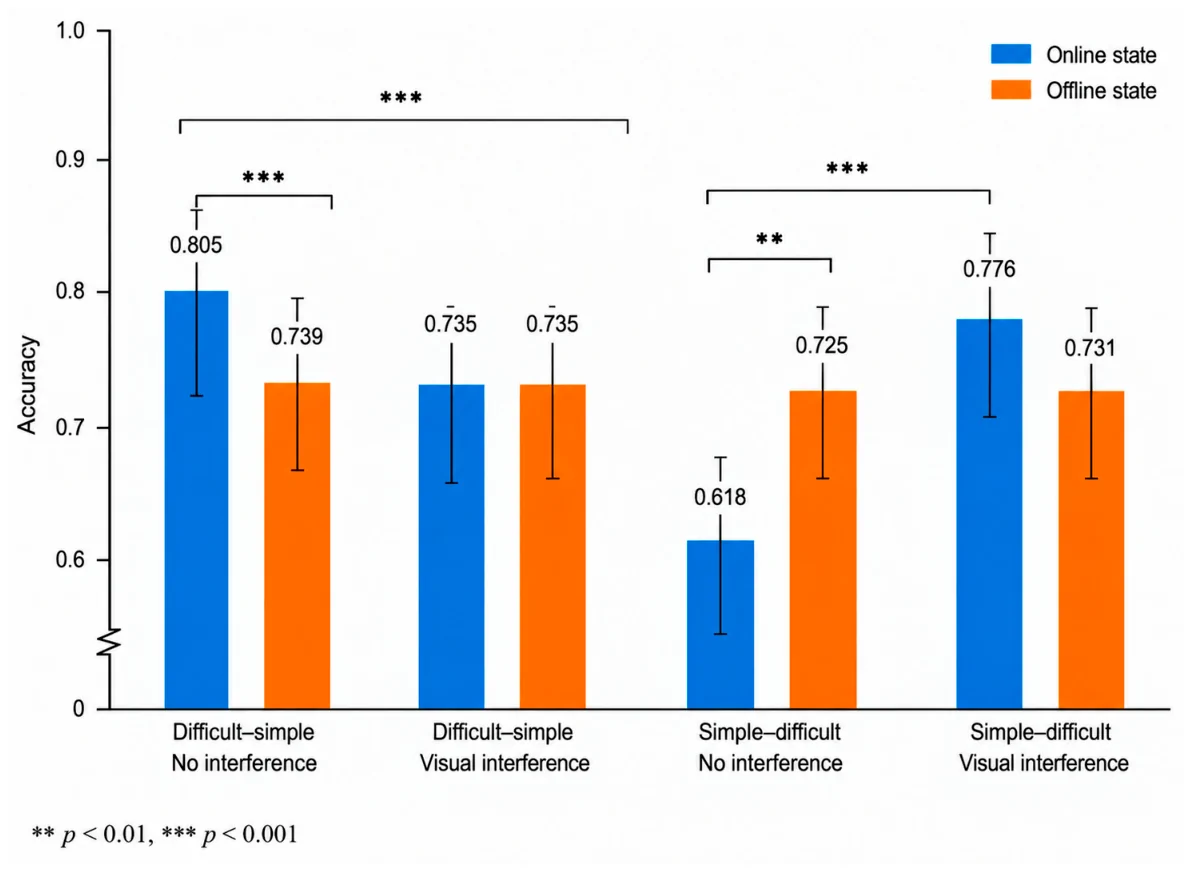
Effects of operationalized working memory state, difficulty sequence, and visual interference on estimation strategy judgment accuracy.

Follow-up contrasts were conducted to examine the source of the three-way interaction. In the online state, visual interference significantly reduced accuracy in the difficult–simple sequence, OR = 0.67, 95% CI [0.51, 0.89], Holm-adjusted *p* = 0.036. In contrast, visual interference significantly increased accuracy in the simple–difficult sequence, OR = 2.15, 95% CI [1.43, 3.23], Holm-adjusted *p* = 0.002. In the offline state, visual interference did not significantly influence accuracy in either the difficult–simple or simple–difficult sequence after correction. Thus, visual interference did not produce a uniform impairment effect but instead showed sequence-dependent modulation in the online state, as shown in Figure 2.

Additional contrasts examined differences between the online and offline states within each difficulty sequence and interference condition. The relative advantage of the two operationalized working memory states was not consistent across contexts, and no general performance advantage was observed for either condition across all experimental conditions. Instead, online and offline state differences varied as a function of difficulty sequence and interference context.

Equation judgment accuracy was examined descriptively as a validation measure and was not included in the primary inferential model. Collapsed across difficulty sequence and visual interference conditions, equation judgment accuracy was 0.893 (SD = 0.074) for online state probes and 0.796 (SD = 0.102) for offline state probes. These results indicate that participants generally retained equation-specific information above chance level, although they do not establish the specific representational format used during task performance.

### 3.4. Discussion

Experiment 2 examined whether visual arithmetic interference modulated estimation strategy processing across task-defined online and offline working memory states and difficulty sequences. The results showed a significant main effect of difficulty sequence, a significant difficulty sequence × visual interference interaction, and, most importantly, a significant three-way interaction among working memory state, difficulty sequence, and visual interference. The simple contrasts clarified this interaction. Within the online state, visual interference reduced accuracy in the difficult–simple sequence but increased accuracy in the simple–difficult sequence. Moreover, without interference, online accuracy was lower for simple–difficult than for difficult–simple sequences. In contrast, visual interference did not significantly affect either difficulty sequence in the offline state. Thus, visual interference did not impose a uniform performance cost; rather, its behavioral consequences depended jointly on the sequential processing context and the task-defined accessibility of the probed information.

This pattern can be interpreted within working memory state accounts that emphasize dynamic priority and accessibility. Information currently prioritized for ongoing behavior is typically more accessible than information maintained outside the immediate focus of attention (Oberauer, 2002; Lewis-Peacock et al., 2012; Olivers et al., 2011; Myers et al., 2017). In the present paradigm, the online state was immediately relevant for the first probe, whereas the offline state had to remain available while another problem was processed before later retrieval. The absence of an overall working memory state main effect indicates that one operationalized working memory state was not uniformly superior to the other. Instead, the significant three-way interaction suggests that the behavioral consequences associated with working memory state priority emerged conditionally, depending on both difficulty sequence and the presence of visual arithmetic interference. This finding is consistent with a dynamic rather than fixed account of working memory state priority: prioritization may alter how information responds to concurrent processing demands without necessarily producing a general performance advantage.

The opposite effects of visual interference across the two online state difficulty sequences are particularly informative. When a difficult problem was followed by a simple problem, visual interference reduced online state strategy judgment accuracy. One possible interpretation is that the additional visual symbolic and arithmetic information competed with resources involved in maintaining or retrieving the currently relevant strategy representation. This interpretation is compatible with evidence that prioritized working memory state representations, despite their enhanced accessibility, remain susceptible to distraction (Z. Zhang & Lewis-Peacock, 2023a, 2023b; Vergauwe et al., 2025). Importantly, however, prioritization did not simply increase vulnerability to distraction: when a simple problem was followed by a difficult problem, the same visual interference manipulation increased online state accuracy. This reversal suggests that interference interacted with the processing context rather than exerting a fixed disruptive effect.

Several mechanisms could potentially account for this improvement, although the present behavioral design cannot distinguish between them. The presence of an additional arithmetic stimulus may have increased attentional engagement, altered the allocation of processing resources, or encouraged participants to maintain the currently relevant strategy information more selectively. Alternatively, the improvement may partly reflect differences in baseline performance, because online accuracy in the simple–difficult/no-interference condition was relatively low. Accordingly, the present results should not be taken as evidence for a specific compensatory control mechanism. More conservatively, they suggest that concurrent distraction can alter the processing of prioritized strategy information in qualitatively different directions depending on the sequential context. This observation extends previous findings that prioritization changes the accessibility of working memory state representations without necessarily insulating them from distraction (Myers et al., 2017; Z. Zhang & Lewis-Peacock, 2023a, 2023b, 2025).

The significant difficulty sequence × visual interference interaction further highlights the role of sequential context. Previous studies have shown that arithmetic strategy execution can vary as a function of the processing demands associated with preceding problems (Uittenhove & Lemaire, 2012, 2013), while computational estimation studies have demonstrated that working memory state updating and task characteristics contribute to strategy selection and execution (Hammerstein et al., 2019, 2021, 2022). In the present experiment, the significant difficulty sequence main effect indicates that performance differed between the two cross-difficulty sequences when averaged across the other experimental factors. More importantly, the sequence × interference interaction shows that this difference itself changed when visual arithmetic distraction was introduced. Together with the three-way interaction, this pattern suggests that sequential context is not merely an additional source of task difficulty; rather, it conditions how concurrent interference is expressed across different levels of working memory state accessibility.

At the same time, the present sequence manipulation does not isolate a residual load mechanism. Difficult–simple and simple–difficult sequences differ simultaneously in the difficulty of the preceding and current problems. Consequently, the observed sequence effects may reflect the difficulty of the currently processed problem, influences carried over from the preceding problem, or their interaction. The present findings therefore support a broader interpretation in terms of sequential processing context, rather than demonstrating residual load from the preceding problem specifically. Future studies could independently manipulate preceding and current problem difficulty, for example, by comparing difficult–simple with simple–simple sequences and simple–difficult with difficult–difficult sequences to determine more precisely how preceding processing demands influence subsequent strategy accessibility.

The relative stability of offline performance provides an additional theoretical contrast. Visual interference did not significantly alter offline state accuracy in either difficulty sequence. One possible interpretation is that information no longer prioritized for the immediate probe may interact differently with newly presented visual symbolic input than currently prioritized information. This possibility is broadly consistent with accounts proposing functional differences in the accessibility and attentional status of prioritized and non-prioritized working memory state representations (Oberauer, 2002; Lewis-Peacock et al., 2012; Olivers et al., 2011). However, the present results do not establish that offline state representations were intrinsically protected from interference. The offline state also differed from the online state in serial position, retention interval, and probe order, and these factors may contribute to the observed pattern. The more defensible conclusion is therefore that visual interference produced stronger context-dependent modulation for the item operationalized as the online state, whereas performance for the working memory state operationalized as the offline state was comparatively stable under the present task conditions.

Several additional limitations should be considered. The distractors were themselves multiplication equations and therefore shared visual symbolic and numerical characteristics with the target stimuli. Consequently, the observed effects cannot be attributed to visual distraction alone; they may also involve competition between arithmetic representations and other task-related processing demands. Furthermore, because the online/offline state manipulation was operationalized through serial position and fixed probe order, the present behavioral differences should not be interpreted as direct evidence for distinct working memory states. Instead, the findings indicate that task-defined differences in accessibility interact with sequential context and concurrent visual arithmetic processing demands.

Despite these limitations, Experiment 2 extends Experiment 1 in an important way. Experiment 1 showed that performance associated with operationalized online and offline states varied across presentation durations, whereas Experiment 2 demonstrates that these accessibility-related differences are also sensitive to concurrent processing context. More broadly, the findings suggest that working memory state priority does not determine arithmetic strategy processing in isolation. Its behavioral consequences emerge through interactions with the difficulty structure of ongoing processing and the characteristics of competing information. Experiment 3 therefore examined whether adding overt articulatory demands to the visual arithmetic distractor would produce a different pattern of modulation across the same operationalized working memory states and difficulty sequences.

## 4. Experiment 3

### 4.1. Experiment 3 Overview and Hypotheses

Experiment 3 further examined whether additional articulatory processing demands would modulate arithmetic estimation strategy processing across the operationalized online and offline working memory states. Building on Experiment 2, in which visual arithmetic distractors were introduced, Experiment 3 required participants to read aloud the distractor equations before completing the probe judgments, thereby increasing verbal and articulatory processing demands (A. Baddeley, 2000; Camos et al., 2009). Because this manipulation involves both vocal production and attentional coordination in addition to articulatory processing, we hypothesized that articulatory interference would differentially influence estimation strategy processing depending on working memory state and difficulty sequence, rather than producing a uniform performance impairment.

### 4.2. Method

#### 4.2.1. Participants

An a priori power analysis was conducted before data collection using G*Power 3.1.9.7 to determine the required sample size for Experiment 3. The analysis used the F-test family with a repeated-measures ANOVA (within-factors) as the design-level framework for the 2 × 2 × 2 within-subject design. The parameters were an effect size of f = 0.25, α = 0.05, statistical power (1 − β) = 0.90, a correlation of 0.50 among repeated measures, and a nonsphericity correction of ε = 1.00. The analysis indicated a minimum required sample size of 20 participants. Because the experimental task was relatively demanding and some data loss was anticipated after application of the predefined performance criteria, we recruited more participants than this minimum requirement to ensure an adequate final sample.

A total of 58 university students were recruited for Experiment 3, including 15 males and 43 females. All participants were aged 18 years or older, right-handed, and reported normal or corrected-to-normal vision, with no amblyopia or color blindness. None had participated in Experiments 1 or 2. The exclusion criteria were specified a priori, before data analysis, and were identical to those used in the previous experiments. Because both the estimation strategy judgment and equation judgment tasks required binary responses, participants were retained only when accuracy was at least 0.50 overall and separately for the online and offline state responses. Sixteen participants did not meet these predefined performance criteria and were excluded. No participants were excluded for other reasons. The final sample consisted of 42 participants. The G*Power analysis was based on a repeated-measures ANOVA framework and therefore served as a design-level estimate of the required sample size. It did not directly model the trial-level binomial GEE used for the primary analyses or specifically estimate statistical power for the three-way interaction. The power analysis should therefore be interpreted as an a priori design-level sample-size reference rather than a direct power analysis of the final GEE model.

All participants provided written informed consent before the experiment and were informed of the experimental procedure, potential risks, task requirements, and their right to withdraw at any time. The study was approved by the university ethics committee.

#### 4.2.2. Materials

The stimuli consisted of two-digit multiplication problems constructed according to the same criteria as in Experiment 2. Problem difficulty was determined based on the unit digits of the two operands. A problem was classified as simple when the unit digits of both operands were smaller than 5, whereas a problem was classified as difficult when both unit digits were larger than 5 or when one unit digit was larger than 5 and the other was smaller than 5. Based on the difficulty of M1 and M2, two cross-difficulty sequence types were defined: difficult–simple and simple–difficult. In the present context, “simple” and “difficult” refer to experimentally defined estimation processing difficulty rather than participants’ spontaneous strategy choices. The distractor equations used in the articulatory interference condition followed the same stimulus construction criteria as the target multiplication problems.

#### 4.2.3. Apparatus and Software

The experiment was programmed and presented using PsychoPy. The apparatus and software were identical to those used in Experiment 1.

#### 4.2.4. Design

Experiment 3 employed a 2 × 2 × 2 within-subjects design, with working memory state (online state vs. offline state), difficulty sequence (difficult–simple vs. simple–difficult), and interference condition (articulatory interference vs. no interference) as within-subject factors. The second presented problem (M2), which was probed first, was operationalized as the online state, whereas the first presented problem (M1), which was probed after M2, was operationalized as the offline state. The dependent variable was accuracy in the estimation strategy consistency judgment task.

The experiment consisted of two blocks, with 60 trial sets in each block, resulting in 120 trial sets per participant. Each trial set followed the sequence M1 → M2 → interference manipulation → T2 → T1 and generated two probe-level responses, yielding 240 probe-level responses per participant. Both M1 and M2 were presented for 1500 ms, following the procedure used in Experiment 2.

Difficulty sequence was defined according to the difficulty of M1 and M2: difficult–simple sequences consisted of a difficult M1 followed by a simple M2, whereas simple–difficult sequences consisted of a simple M1 followed by a difficult M2. Articulatory interference and no-interference trials were equally represented and randomly intermixed across participants. The two difficulty sequences were also randomly presented across trials.

The two judgment types were randomly intermixed within each block, with estimation strategy judgment trials accounting for 70% of trials and equation judgment trials accounting for 30% of trials. The construction of same/different responses and the generation of estimation strategy and equation judgments followed the procedures described in Experiment 1. The three rounding strategies (rounding down, rounding up, and mixed rounding) were approximately balanced across trials.

The interference condition differed from Experiment 2 in that participants were required to read aloud the visually presented distractor equation before proceeding to the probe phase. Thus, the articulatory interference manipulation introduced additional articulatory processing demands beyond the visual and arithmetic processing demands present in Experiment 2.

#### 4.2.5. Procedure

The practice phase and strategy training procedure were identical to those in Experiment 1 and Experiment 2.

Each formal trial began with a fixation cross presented at the center of the screen for 500 ms. The first multiplication problem (M1) was then presented for 1500 ms, followed by a 500 ms black and white mosaic mask. The second multiplication problem (M2) was subsequently presented for 1500 ms and followed by another identical 500 ms mask.

After the second mask, the trial diverged according to the interference condition. In the articulatory interference condition, an irrelevant two-digit multiplication problem was presented at the center of the screen for 1500 ms. Participants were instructed to read the distractor equation aloud while viewing it. The distractor equations followed the same stimulus construction criteria as the target multiplication problems, and were randomly generated while avoiding identity with M1, M2, T1, or T2 within the same trial. In the no-interference condition, a blank screen was presented for 1500 ms instead, thereby matching the duration between the second mask and the probe phase across conditions.

Following the interference manipulation, two probes were presented sequentially in the fixed order T2 followed by T1. The articulatory interference and no-interference trials were equally represented and randomly intermixed across participants. The three factors were fully randomized at the trial level. The probe procedure and response requirements were identical to those used in the previous experiments. All responses were made using a binary same/different judgment.

Each probe remained on the screen until a response was made or until the 5000 ms response deadline was reached. Responses exceeding the deadline were coded as incorrect. Participants completed two experimental blocks, and the order of trials was randomized across participants.

#### 4.2.6. Data Analysis

All analyses were conducted in Python 3.9.13 using pandas 1.4.4, NumPy 1.21.5, SciPy 1.9.1, statsmodels 0.13.2, and patsy 0.5.2. Trial-level accuracy in the estimation strategy judgment task was analyzed using a population-averaged generalized estimating equation (GEE) with a binomial distribution and logit link. Working memory state (online state vs. offline state), difficulty sequence (difficult–simple vs. simple–difficult), articulatory interference (interference vs. no interference), and their interactions were included as fixed effects, with the participant treated as the clustering variable and an exchangeable working correlation structure used to account for repeated observations.

Omnibus effects were evaluated using Type III Wald χ^2^ tests. Significant interactions were followed by pairwise contrasts, which were expressed as odds ratios (ORs) with 95% confidence intervals. Holm correction was applied to post hoc comparisons. Equation judgment accuracy was summarized descriptively and was not included in the primary inferential model.

### 4.3. Results

Of the 58 participants represented in the raw data archive, 42 met the inclusion criterion that their online state strategy, offline state strategy, online state equation, and offline state equation accuracy were each at least 50%. The primary analysis comprised 7056 trial-level binary strategy judgment responses.

Trial-level accuracy was analyzed using a population-averaged generalized estimating equation (GEE) with a binomial distribution and logit link. Working memory state (online state vs. offline state), difficulty sequence (simple–difficult vs. difficult–simple), articulatory interference (interference vs. no interference), and their interactions were included as fixed effects. The participant was treated as the clustering variable, with an exchangeable working correlation structure and empirical sandwich robust standard errors. The Type III Wald tests showed a significant main effect of articulatory interference, Wald χ^2^(1) = 15.35, *p* < 0.001, OR = 0.75, 95% CI [0.64, 0.86], indicating that reading aloud the arithmetic distractor generally reduced estimation strategy judgment accuracy. The interaction between working memory state and difficulty sequence was significant, Wald χ^2^(1) = 12.59, *p* < 0.001, ratio of odds ratios = 1.41, 95% CI [1.16, 1.70]. This interaction was further qualified by a significant three-way interaction among working memory state, difficulty sequence, and articulatory interference, Wald χ^2^(1) = 3.96, *p* = 0.047, ratio of odds ratios = 0.71, 95% CI [0.50, 0.99]. In contrast, the main effects of working memory state (Wald χ^2^(1) = 3.21, *p* = 0.073) and difficulty sequence (Wald χ^2^(1) = 0.44, *p* = 0.507) were not significant.

**Figure 3:**
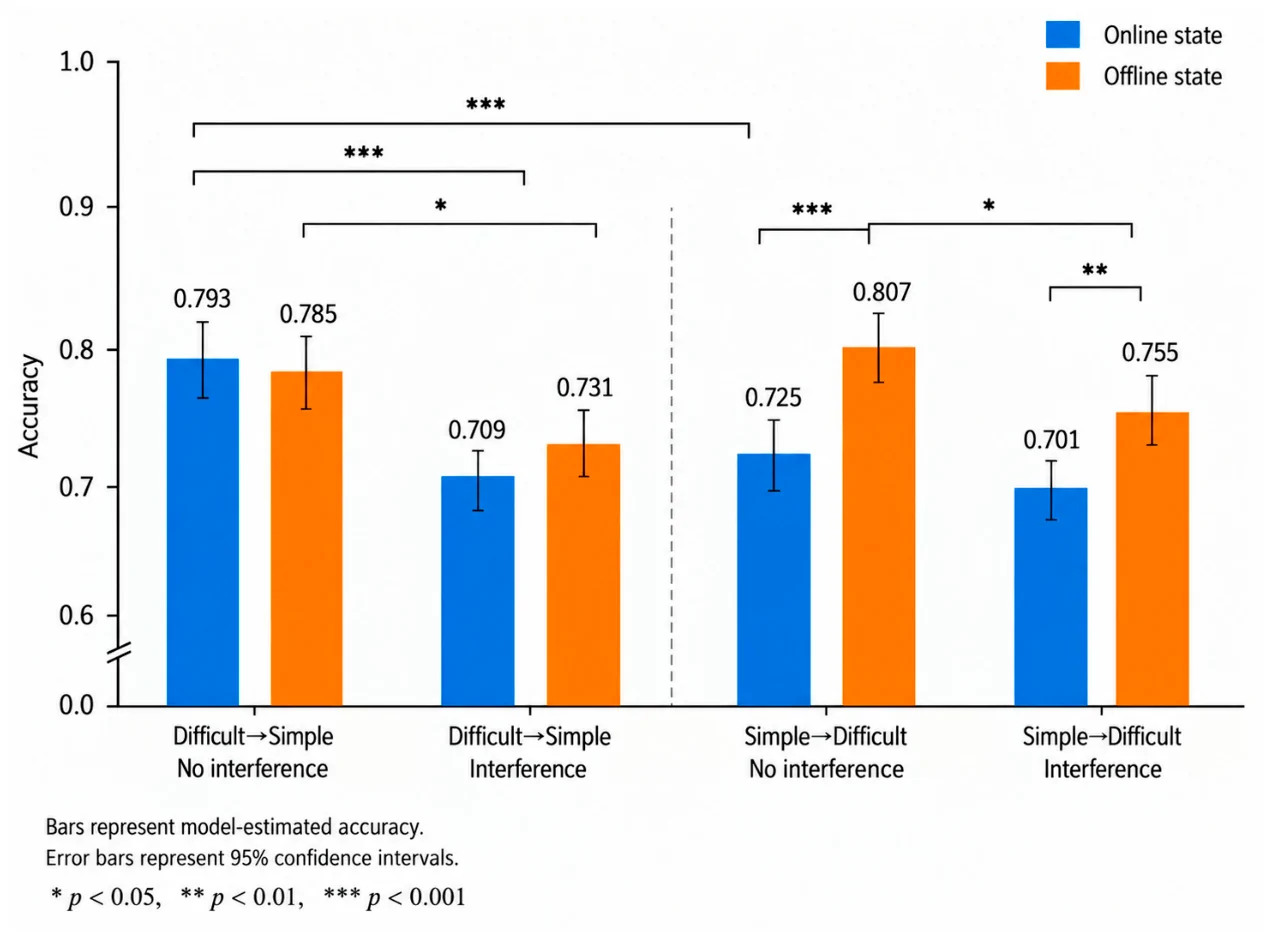
Effects of operationalized working memory state, difficulty sequence, and articulatory interference on estimation strategy judgment accuracy.

The model-estimated accuracy across the eight experimental conditions is presented in Figure 3. In the online state, accuracy for the simple–difficult sequence was 0.725 (95% CI [0.663, 0.779]) in the no-interference condition and 0.701 (95% CI [0.660, 0.739]) in the articulatory interference condition. For the difficult–simple sequence, online state accuracy was 0.793 (95% CI [0.750, 0.831]) without interference and decreased to 0.709 (95% CI [0.661, 0.753]) with interference. In the offline state, accuracy was relatively stable across interference conditions. Specifically, accuracy for the simple–difficult sequence was 0.807 (95% CI [0.758, 0.848]) without interference and 0.755 (95% CI [0.708, 0.796]) with interference, whereas accuracy for the difficult–simple sequence was 0.785 (95% CI [0.733, 0.829]) without interference and 0.731 (95% CI [0.679, 0.776]) with interference.

Follow-up contrasts were conducted to examine the source of the significant interactions. Holm-adjusted comparisons showed that, within the simple–difficult sequence, online state accuracy was lower than offline state accuracy both without articulatory interference (OR = 0.63, 95% CI [0.46, 0.86], raw *p* = 0.003, Holm-adjusted *p* = 0.013) and with articulatory interference (OR = 0.76, 95% CI [0.61, 0.95], raw *p* = 0.015, Holm-adjusted *p* = 0.046). However, no significant online and offline state differences were observed within the difficult–simple sequence after correction (adjusted *p* ≥ 0.612).

The effects of articulatory interference were further examined within each working memory state × difficulty sequence combination. Articulatory interference significantly reduced accuracy in the online state difficult–simple condition (OR = 0.64, 95% CI [0.52, 0.78], raw *p* < 0.001, Holm-adjusted *p* < 0.001), the offline state simple–difficult condition (OR = 0.74, 95% CI [0.58, 0.93], raw *p* = 0.011, Holm-adjusted *p* = 0.021), and the offline state difficult–simple condition (OR = 0.74, 95% CI [0.61, 0.91], raw *p* = 0.005, Holm-adjusted *p* = 0.014). In contrast, articulatory interference did not significantly affect online state accuracy in the simple–difficult condition (OR = 0.89, 95% CI [0.68, 1.16], Holm-adjusted *p* = 0.389). These comparisons are illustrated in Figure 3.

Difficulty sequence contrasts were additionally examined within each working memory state and interference context. In the absence of articulatory interference, online state accuracy was lower for the simple–difficult sequence than for the difficult–simple sequence (OR = 0.69, 95% CI [0.57, 0.83], raw *p* < 0.001, Holm-adjusted *p* < 0.001). The remaining sequence contrasts were not significant after correction (adjusted *p* ≥ 0.661).

Equation judgment accuracy was examined descriptively as a validation measure and was not included in the primary inferential model. Accuracy was higher for online state probes (M = 0.864, SD = 0.080) than for offline state probes (M = 0.729, SD = 0.095).

### 4.4. Discussion

Experiment 3 examined whether increasing verbal and articulatory processing demands through an articulatory interference manipulation would further modulate estimation strategy processing across operationalized online and offline working memory states. The results showed that articulatory interference significantly reduced overall strategy judgment accuracy, and its influence varied as a function of working memory state and difficulty sequence. Specifically, articulatory interference reduced accuracy in the online state difficult–simple condition and in both offline state sequence conditions, whereas the online state simple–difficult condition was relatively unaffected. These findings indicate that introducing additional concurrent processing demands altered the accessibility of estimation strategy information, although the magnitude of this effect depended on the task context. More broadly, the pattern suggests that the behavioral consequences of concurrent interference depend not only on the amount of additional processing imposed, but also on the current functional accessibility of the information being maintained.

The overall interference cost observed in Experiment 3 is consistent with previous research showing that concurrent articulation and speech-related processing can disrupt short-term retention and ongoing cognitive processing by competing with verbal or articulatory resources (A. Baddeley, 2000; Camos et al., 2009). In particular, articulatory suppression and related read-aloud manipulations have been shown to interfere with the maintenance of information that relies on verbal coding or rehearsal processes (Larsen & Baddeley, 2003). However, the present manipulation should not be interpreted as a pure phonological interference manipulation. Participants were required to read aloud arithmetic distractors rather than perform a selective articulatory suppression task; therefore, the observed effects may reflect a combination of articulatory production, attentional coordination, and the concurrent processing of arithmetic information. One plausible interpretation is that overt articulation increased the number of processing operations that had to be coordinated during the retention interval. Under this account, participants were required not only to maintain task-relevant arithmetic and strategy information, but also to process and vocalize a competing arithmetic stimulus before retrieval. This additional coordination demand may have reduced the efficiency with which previously encoded strategy information could be accessed at probe, without implying that the underlying representation itself was lost.

This interpretation is consistent with working memory frameworks in which maintenance depends on the balance between attentional refreshing and domain-specific rehearsal processes (Camos et al., 2009). If articulatory activity occupies resources that could otherwise contribute to maintaining or refreshing strategy-related information, retrieval may become less reliable when the probe is presented. At the same time, because the distractor was itself arithmetic, the effect may also reflect competition at the level of numerical and strategy-related processing. The present experiment cannot determine the relative contribution of these possibilities, but it indicates that adding overt articulation changes the processing environment in a way that is behaviorally consequential for estimation strategy judgments.

The interaction between working memory state and difficulty sequence further suggests that the effect of articulatory interference was not uniform across task contexts. In the absence of interference, online state accuracy was lower for the simple–difficult sequence than for the difficult–simple sequence, whereas this sequence difference was not observed under most other conditions. One possible interpretation is that the accessibility of estimation strategy information depends on the relationship between the currently processed item and the preceding processing context. Previous research on arithmetic strategy selection has shown that strategy execution is influenced by sequential problem context and by the cognitive demands imposed by preceding problems (Uittenhove & Lemaire, 2012, 2013). Similarly, studies on computational estimation have demonstrated that working memory state updating and task demands influence the selection and execution of estimation strategies (Hammerstein et al., 2019, 2021, 2022). The disappearance of this sequence difference under articulatory interference may indicate that additional concurrent processing demands reduce the behavioral expression of differences associated with sequential context. One possibility is that when the retention interval already contains substantial articulatory and attentional demands, the relative contribution of the preceding or current difficulty configuration becomes less distinct because multiple sources of processing demand converge before probe retrieval. However, because the present design manipulated difficulty sequence rather than independently manipulating previous problem difficulty and current problem difficulty, these results cannot be interpreted as direct evidence for residual load mechanisms.

The present findings also provide further evidence that prioritization in working memory does not necessarily imply complete protection from interference. Working memory state theories distinguish between information that is currently prioritized for processing and information that remains available but outside the immediate focus of attention (Oberauer, 2002; Lewis-Peacock et al., 2012; Myers et al., 2017). The present online and offline state manipulation was designed to functionally approximate these differences in accessibility. Although articulatory interference produced stronger effects in some online states, the pattern did not indicate that online state information was universally more vulnerable or that offline state information was universally protected. Instead, interference sensitivity depended on the combination of working memory state, difficulty sequence, and the specific demands introduced by the distractor. This pattern is theoretically important because it argues against the simple assumption that prioritized information is necessarily protected from interference. Priority may enhance immediate accessibility, but that same accessibility may also require continued coordination with ongoing task demands. Conversely, information that is less immediately prioritized may remain retrievable but may become more dependent on successful reaccess after concurrent processing. The present results therefore suggest that accessibility and interference susceptibility are related in a context-sensitive rather than monotonic manner.

The offline state effects are especially informative in this respect. Articulatory interference reduced offline accuracy in both difficulty sequences, whereas visual interference in Experiment 2 left offline performance comparatively stable. One possible interpretation is that the additional read-aloud requirement placed greater demands on processes needed to maintain or later retrieve information that was no longer immediately prioritized. Because the offline item had to survive the processing of M2, the distractor, and the first probe before T1 was presented, additional articulatory demands may have increased the difficulty of later access to that retained information. Importantly, this does not imply that the offline state representation itself was disrupted or erased; rather, the observed decline may reflect reduced retrieval readiness or less efficient reactivation at the time of probing.

The contrast between Experiments 2 and 3 therefore provides a broader theoretical insight. Visual arithmetic interference alone produced highly context-dependent effects, including both impairment and improvement in the online condition, whereas adding overt articulation resulted in a more consistent reduction in accuracy. This difference suggests that interference effects in arithmetic estimation cannot be understood solely in terms of stimulus modality. Instead, the number and type of concurrent operations that must be coordinated—including visual symbolic processing, arithmetic processing, read aloud, and attentional control requirements—may jointly determine how accessible strategy information remains. In this sense, Experiment 3 extends the interference account from a modality-based explanation to a processing demand account.

Several limitations should be considered when interpreting these findings. First, the read-aloud distractors shared arithmetic and visual characteristics with the target equations. Therefore, the observed effects cannot be attributed solely to articulatory-based interference; competition between arithmetic representations and general task switching or attentional demands may also contribute. Second, as in the previous experiments, the online and offline states differed in serial position, retention interval, and probe order. Thus, the current findings demonstrate context-dependent behavioral differences between task-defined online and offline states rather than directly establishing distinct working memory states. Accordingly, the proposed explanations involving resource competition, reduced retrieval readiness, or attentional coordination should be treated as theoretically plausible accounts of the observed pattern rather than as directly demonstrated mechanisms.

Taken together, Experiment 3 extends the findings of Experiment 2 by showing that increasing concurrent verbal and articulatory demands produces a different pattern of modulation compared with visual arithmetic interference. Whereas visual interference in Experiment 2 produced context-dependent changes in performance, articulatory interference generated a more consistent reduction in strategy judgment accuracy. These findings suggest that the accessibility of estimation strategy information is sensitive to the type of concurrent processing demands imposed during retention and retrieval. More broadly, they indicate that arithmetic strategy processing depends on the interaction between the functional accessibility of maintained information and the processing operations that occur before retrieval. This perspective moves beyond a simple distinction between “visual” and “verbal” interference and instead emphasizes how concurrent task demands reshape the accessibility of strategy information within the working memory state. Future work should further disentangle the contributions of articulatory and attentional processes to better characterize how different forms of interference influence working-memory-state-guided arithmetic strategy processing.

## 5. General Discussion

The present study investigated how working memory accessibility influences arithmetic estimation strategy processing across different task contexts. Across three experiments, we examined whether estimation strategy information associated with task-defined online and offline working memory states showed different behavioral patterns over time and under different interference demands. Rather than treating online and offline states as fixed storage states, the present study used these conditions as functional operationalizations of differences in information priority and accessibility. The findings demonstrate that estimation strategy processing is dynamically modulated by the interaction between working memory state, temporal availability, sequential difficulty context, and concurrent processing demands. More broadly, the findings suggest that arithmetic strategy processing depends not only on how much information can be maintained in working memory state, but also on when that information is prioritized, when it must be retrieved, and what competing operations occur during the retention interval.

### 5.1. Dynamic Accessibility of Estimation Strategy Information

Across experiments, the accessibility of estimation strategy information was not static but varied according to the current processing context. Experiment 1 showed that the two operationalized working memory states exhibited different performance patterns as presentation duration changed. When sufficient encoding time was available, performance between the two conditions converged, suggesting that additional processing time can influence later accessibility of previously presented information. One possible interpretation is that processing time changes the relative accessibility of prioritized and subsequently retrieved information in different ways: the initial advantage of immediately relevant information may be transient, whereas additional encoding time may improve later access to information that must be retained across intervening processing. Experiments 2 and 3 further demonstrated that concurrent processing demands altered these patterns, indicating that the accessibility of estimation strategy information depends not only on the temporal availability of information but also on the demands imposed during retention and retrieval.

These findings are consistent with contemporary views of working memory as a flexible system in which information varies in priority, accessibility, and attentional status rather than simply being either stored or absent (Oberauer, 2002; Cowan, 2005; Lewis-Peacock et al., 2012; Myers et al., 2017). Information that is currently prioritized may receive enhanced accessibility, whereas information outside the immediate focus of attention may remain available for subsequent retrieval. The present results extend this perspective by showing that such differences in accessibility are behaviorally relevant during arithmetic strategy processing, a domain that requires maintaining numerical information while selecting and applying estimation strategies (Imbo & Vandierendonck, 2007; Hammerstein et al., 2019, 2021). Thus, the present findings extend accessibility-based accounts of working memory state into arithmetic strategy processing by showing that priority is behaviorally meaningful even when the task requires transformation and evaluation of numerical information rather than simple maintenance.

### 5.2. Working Memory Accessibility and Arithmetic Strategy Processing

The present findings also contribute to understanding how working memory processes support arithmetic estimation strategies. Computational estimation requires individuals to maintain relevant numerical information, select an appropriate transformation strategy, and monitor the relationship between approximate and exact representations. Previous research has demonstrated that strategy selection and execution depend on working memory updating ability, executive resources, and task characteristics (Fürst & Hitch, 2000; Imbo & Vandierendonck, 2007; Hammerstein et al., 2021, 2022). The current findings further suggest that the accessibility of strategy-related information can be influenced by the temporal status of information and by additional processing demands introduced during the task. This suggests that working memory state may contribute to estimation not only by providing a limited resource for maintaining operands and rules, but also by dynamically regulating which strategy-related representation is most readily available at a given moment.

Importantly, the present results do not suggest that online state information is always superior or that offline state information is always impaired. Instead, the relative accessibility of these task-defined conditions varied depending on the experimental context. For example, interference effects differed between visual arithmetic distraction and articulatory distraction, indicating that the consequences of reduced accessibility depend on the specific demands imposed on the cognitive system. This pattern is consistent with the view that working memory operates through dynamic prioritization rather than through a simple distinction between active and inactive representations (Olivers et al., 2011; Z. Zhang & Lewis-Peacock, 2023a, 2023b). Accordingly, prioritization appears better understood as a flexible processing advantage whose behavioral expression depends on concurrent task demands, rather than as a fixed guarantee of superior performance or resistance to interference.

### 5.3. Interference Effects Depend on Concurrent Processing Demands

A major finding of the present study is that interference effects were not uniform. Instead, different types of interference produced distinct patterns of modulation. In Experiment 2, visual arithmetic distractors produced context-dependent effects, with interference effects varying according to working memory state and difficulty sequence. The reversal of the interference effect across the two online difficulty sequences is particularly informative because it indicates that distraction can either impair or facilitate performance depending on the current sequential context. This suggests that concurrent input interacts with the ongoing organization of strategy processing rather than simply consuming a fixed amount of working memory capacity. In Experiment 3, introducing overt vocalization generated a more consistent reduction in strategy judgment accuracy, suggesting that additional verbal and articulatory demands can place further constraints on strategy processing.

These findings indicate that interference should not be understood as a single mechanism. Visual arithmetic distractors may compete with target processing through overlapping visual, numerical, and symbolic representations, whereas articulatory interference additionally introduces articulatory and response production demands (A. Baddeley, 2000; Camos et al., 2009). Previous research has shown that concurrent speech-related processing can interfere with information maintenance by engaging verbal working memory resources (Larsen & Baddeley, 2003). The current findings extend this perspective by showing that the behavioral consequences of interference depend on both the nature of the distractor and the accessibility demands of the information being processed. The contrast between Experiments 2 and 3 therefore supports a processing demand interpretation: interference effects may become more widespread as the concurrent task requires coordination across visual symbolic, arithmetic, articulatory, and attentional processes.

### 5.4. Theoretical Contribution and Interpretation Boundary

The present study provides behavioral evidence that working memory accessibility during arithmetic strategy processing is dynamically influenced by temporal availability and concurrent processing demands. Its theoretical contribution therefore lies less in demonstrating discrete storage states than in showing that task-defined differences in priority and accessibility have measurable consequences for arithmetic strategy processing. However, several conceptual boundaries should be emphasized. Although online and offline states were motivated by theories of working memory priority and accessibility (Oberauer, 2002; Lewis-Peacock et al., 2012), the current paradigm does not directly measure distinct neural storage states (Christophel et al., 2017). Behavioral differences between the two conditions may also reflect differences in serial position, retention interval, and probe order. Therefore, the present findings support a functional distinction in accessibility rather than demonstrate separate representational states.

Similarly, the difficulty sequence manipulation should be interpreted as a contextual manipulation of sequential processing demands rather than direct evidence for residual load mechanisms. Previous studies have shown that arithmetic strategy execution can be influenced by preceding problem characteristics and sequential context (Uittenhove & Lemaire, 2012, 2013), but the current design did not independently manipulate preceding and current problem difficulty. The present results nevertheless suggest that sequential context is theoretically relevant because the effect of interference changed depending on the arrangement of difficulty across successive problems. Thus, sequential context may shape how working memory accessibility is expressed even if the present design cannot isolate the specific contribution of residual load. Future studies should therefore isolate these components more directly.

### 5.5. Limitations and Future Directions

Several limitations provide directions for future research. First, the serial probe paradigm used in the present study inevitably couples working memory state with serial position and probe order. Future studies could independently manipulate information priority, retention interval, and probe timing to more directly examine accessibility changes while minimizing these confounds. Combining behavioral paradigms with neurophysiological measures, such as EEG or fMRI, may further clarify whether behavioral differences correspond to distinct neural states or reflect changes in attentional priority and retrieval accessibility.

Second, the interference manipulations used here involved arithmetic distractors, and therefore combined multiple processing demands. Future research could systematically separate visual, articulatory, and arithmetic components by using matched distractors that differ only in specific cognitive requirements. Such designs would help determine which aspects of interference are most influential in modulating strategy accessibility. They would also allow future work to test whether the broader disruption observed under articulatory interference reflects read-aloud demands specifically or the cumulative burden of coordinating several concurrent processing operations.

Third, the present study focused on estimation strategy processing in multiplication tasks. Future research should examine whether similar accessibility dynamics occur across different arithmetic operations, age groups, and levels of mathematical expertise. Such extensions would help clarify the generality of working-memory-guided strategy regulation in numerical cognition. If similar priority-dependent patterns emerge across other operations and populations, this would suggest that dynamic accessibility is a broader principle of strategic numerical cognition rather than a feature specific to multiplication estimation.

In conclusion, the present study demonstrates that arithmetic estimation strategy processing is dynamically shaped by the accessibility of maintained information and by concurrent processing demands. While the findings do not establish distinct working memory storage states, they reveal systematic behavioral differences between task-defined online and offline states and provide new insights into how working memory accessibility supports flexible strategy use during complex cognitive processing. Taken together, the three experiments suggest that arithmetic strategy performance emerges from the interaction between information priority, temporal availability, sequential context, and concurrent processing demands, providing a broader framework for understanding how working memory supports flexible strategy regulation.

## 6. Conclusions

The present study investigated how working memory states, operationalized as online and offline states, relate to arithmetic estimation strategy processing under different temporal and interference contexts. Across three experiments, the findings consistently showed that estimation strategy information was not processed in a fixed manner but varied according to its task-defined accessibility, sequential context, and concurrent processing demands.

Together, these results indicate that working memory states are associated with dynamic differences in the accessibility and use of arithmetic strategy information under varying task demands. Importantly, the present findings should be interpreted as evidence for functional differences between task-defined online and offline working memory states rather than direct evidence for distinct storage states or specific neural mechanisms. The observed behavioral differences may reflect changes in information priority, accessibility, and retrieval demands, which are also influenced by serial position, retention interval, and probe order inherent in the present paradigm.

## Data Availability

The original data supporting the findings reported in this study are available from the corresponding author upon reasonable request. The original data used in the present study form part of an ongoing doctoral dissertation and are still being retained for the completion of the associated doctoral research. Therefore, the data are not currently deposited in a public repository.
